# *N*-(1,5-Dimethyl-3-oxo-2-phenyl-2,3-di­hydro-1*H*-pyrazol-4-yl)-2-phenyl­acetamide

**DOI:** 10.1107/S1600536813029590

**Published:** 2013-11-06

**Authors:** Manpreet Kaur, Jerry P. Jasinski, Brian J. Anderson, H. S. Yathirajan, B. Narayana

**Affiliations:** a Department of Studies in Chemistry, University of Mysore, Manasagangotri, Mysore 570 006, India; b Department of Chemistry, Keene State College, 229 Main Street, Keene, NH 03435-2001, USA; c Department of Studies in Chemistry, Mangalore University, Mangalagangotri 574 199, India

## Abstract

The title compound, C_19_H_19_N_3_O_2_, crystallizes with two independent mol­ecules (*A* and *B*) in the asymmetric unit. In mol­ecule *A*, the pyrazole ring adopts a slightly disordered half-chair conformation while in *B* it is planar [r.m.s. deviation = 0.0386 (15) Å]. The dihedral angle between the mean planes of the two phenyl rings is 56.2 (8) in *A* and 38.2 (3)° in *B*. The *N*-phenyl substituent on the pyrazole ring is twisted by 46.5 (2) in *A* and 58.6 (4)° in *B* while the extended phenyl ring is twisted by 82.2 (8) in *A* and 87.5 (9)° in *B*. The mean plane of the amide group forms an angle of 74.8 (3) in *A* and 67.7 (1)° in *B* with respect to the phenyl ring. In addition, the amide group is rotated by 51.4 (1) in *A* and 53.6 (2)° in *B* from the the mean plane of the pyrazole ring. In the crystal, the two molecules are linked *via* N—H⋯O hydrogen bonds, supported by weak C—H⋯O inter­actions, forming dimers enclosing an *R*_2_^2^(10) ring motif. The dimers are linked *via* C—H⋯O inter­actions, forming a three-dimensional structure.

## Related literature

For the structural similarity of *N*-substituted 2-aryl­acetamides to the lateral chain of natural benzyl­penicillin, see: Mijin *et al.* (2008 ▶). For the coordination abilities of amides, see: Wu *et al.* (2008 ▶, 2010 ▶). For the pharmaceutical, insecticidal and non-linear properties of pyrazoles, see: Chandrakantha *et al.* (2013 ▶); Cheng *et al.* (2008 ▶); Hatton *et al.* (1993 ▶); Liu *et al.* (2010 ▶). For related structures, see: Fun *et al.* (2011*a* ▶,*b* ▶, 2012 ▶); Butcher *et al.* (2013*a* ▶,*b* ▶). For puckering parameters, see Cremer & Pople (1975 ▶). For standard bond lengths, see: Allen *et al.* (1987 ▶). 
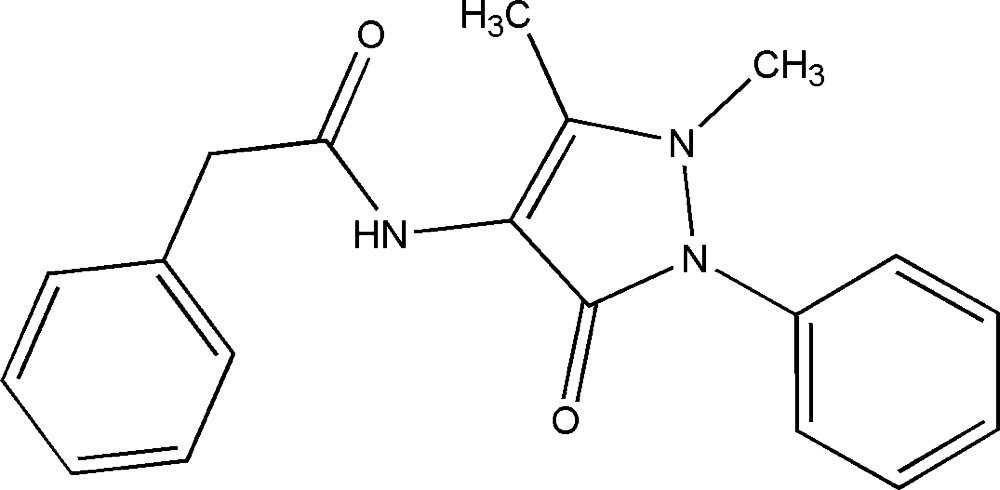


## Experimental

### 

#### Crystal data

C_19_H_19_N_3_O_2_
*M* *_r_* = 321.37
Triclinic, 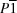

*a* = 10.1258 (7) Å
*b* = 10.4671 (8) Å
*c* = 17.8888 (12) Å
α = 100.833 (6)°
β = 92.527 (5)°
γ = 116.812 (7)°
*V* = 1643.9 (2) Å^3^
*Z* = 4
Cu *K*α radiation
μ = 0.69 mm^−1^
*T* = 173 K
0.48 × 0.32 × 0.26 mm

#### Data collection

Agilent Xcalibur (Eos, Gemini) diffractometer
Absorption correction: multi-scan (*CrysAlis PRO* and *CrysAlis RED*; Agilent, 2012 ▶) *T*_min_ = 0.876, *T*_max_ = 1.000
10216 measured reflections
6333 independent reflections
5485 reflections with *I* > 2σ(*I*)
*R*_int_ = 0.037

#### Refinement

*R*[*F* ^2^ > 2σ(*F* ^2^)] = 0.046
*wR*(*F* ^2^) = 0.130
*S* = 1.04
6333 reflections
438 parameters
H-atom parameters constrained
Δρ_max_ = 0.30 e Å^−3^
Δρ_min_ = −0.23 e Å^−3^

### 

Data collection: *CrysAlis PRO* (Agilent, 2012 ▶); cell refinement: *CrysAlis PRO*; data reduction: *CrysAlis RED* (Agilent, 2012 ▶); program(s) used to solve structure: *SUPERFLIP* (Palatinus & Chapuis, 2007 ▶); program(s) used to refine structure: *SHELXL2012* (Sheldrick, 2008 ▶); molecular graphics: *OLEX2* (Dolomanov *et al.*, 2009 ▶); software used to prepare material for publication: *OLEX2*.

## Supplementary Material

Crystal structure: contains datablock(s) I. DOI: 10.1107/S1600536813029590/hg5356sup1.cif

Structure factors: contains datablock(s) I. DOI: 10.1107/S1600536813029590/hg5356Isup2.hkl

Supplementary material file. DOI: 10.1107/S1600536813029590/hg5356Isup3.cml

Additional supplementary materials:  crystallographic information; 3D view; checkCIF report

## Figures and Tables

**Table 1:** Hydrogen-bond geometry (Å, °)

*D*—H⋯*A*	*D*—H	H⋯*A*	*D*⋯*A*	*D*—H⋯*A*
N1*A*—H1*A*⋯O2*B*	0.86	1.97	2.8292 (16)	173
C14*A*—H14*A*⋯O1*A* ^i^	0.93	2.55	3.454 (2)	165
N1*B*—H1*B*⋯O2*A*	0.86	1.98	2.8115 (16)	163
C2*B*—H2*BA*⋯O1*B* ^ii^	0.97	2.55	3.4239 (19)	150
C4*B*—H4*B*⋯O1*B* ^ii^	0.93	2.72	3.487 (2)	141
C8*B*—H8*B*⋯O2*A*	0.93	2.57	3.404 (2)	150
C14*B*—H14*B*⋯O1*A* ^iii^	0.93	2.70	3.398 (2)	132

Symmetry codes: (i) 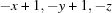; (ii) 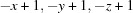; (iii) 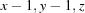.

**Fig. 1.**
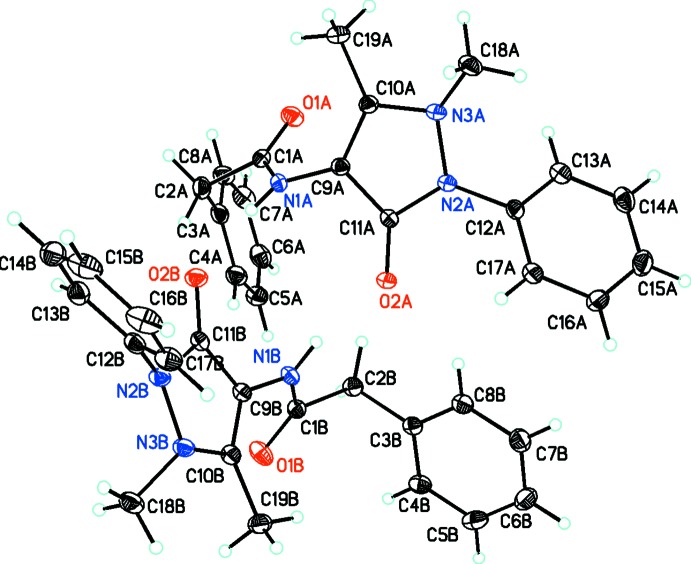
ORTEP drawing of (I) (C19H19N3O2) showing the labeling scheme of molecules A and B with 30% probability displacement ellipsoids.

**Fig. 2.**
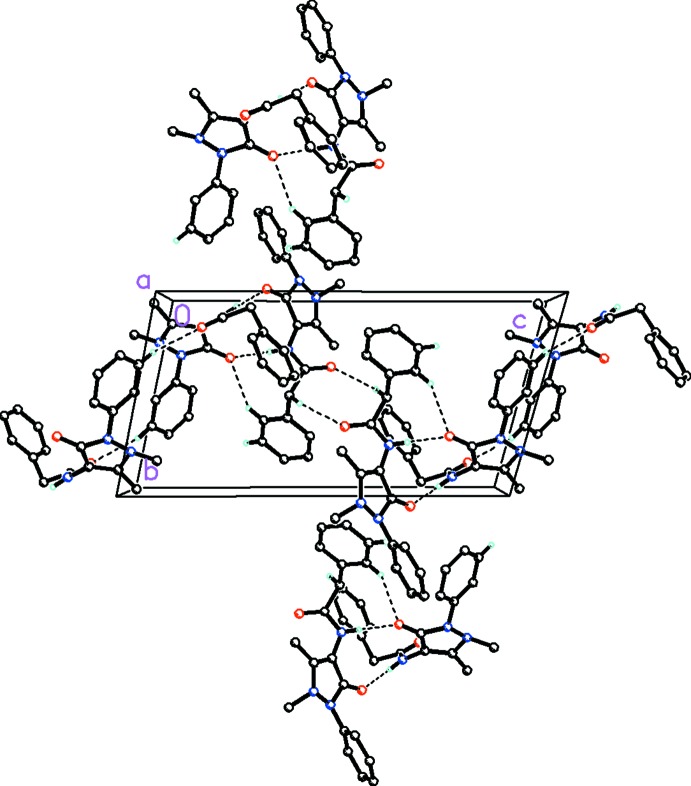
Molecular packing for (I) viewed along the a axis. Dashed lines indicate N—H···O intermolecular hydrogen bonds supported by a weak C—H···O intermolecular interactions link the molecules into dimers forming R22(10) graph set motifs. Also, weak C—H···O intermolecular interactions are observed which interlink the dimers and influence the crystal packing. H atoms not involved in hydrogen bonding have been removed for clarity.

**Fig. 3.**
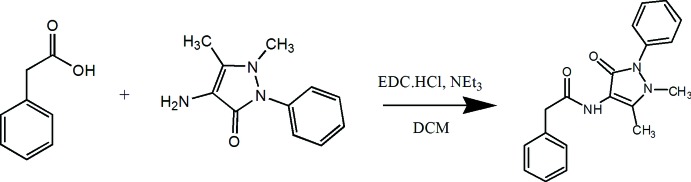
Synthesis scheme of (I).

## supplementary crystallographic information

### 1. Comment

N-Substituted 2-arylacetamides are biologically active compounds because of their structural similarity to the lateral chain of natural benzylpenicillin (Mijin *et al.*, 2008). Amides are also used as ligands due to their excellent coordination abilities (Wu *et al.*, 2008, 2010). In a variety of biological heterocyclic compounds, N-pyrazole derivatives are of great interest because of their chemical and pharmaceutical properties (Cheng *et al.*, 2008). Some of the N-pyrazole derivatives have been found to exhibit good insecticidal activities (Hatton *et al.*, 1993), antifungal activities (Liu *et al.*, 2010) and non-linear optical properties (Chandrakantha *et al.*, 2013). Crystal structures of some related acetamide and pyrazole derivatives are : N-(4-Bromophenyl)-2-(naphthalen-1-yl) acetamide, N-(3,5-Dichlorophenyl)-2-(naphthalen-1-yl)acetamide, N-(1,5-Dimethyl-3-oxo-2-phenyl-2,3-dihydro-1H-pyrazol-4-yl)-2- [4-(methylsulfanyl)phenyl]acetamide, (Fun *et al.*, 2011*a*,*b*, 2012), 2-(2,4-Dichlorophenyl)-N-(1,5-dimethyl-3-oxo-2- phenyl-2,3-dihydro-1H-pyrazol-4-yl)acetamide, 2-(2,6-dichloro phenyl)-N-(1,5-dimethyl-3-oxo-2-phenyl-2,3-dihydro-1H-pyrazol- 4-yl)acetamide (Butcher *et al.*, 2013*a*,*b*) have been reported. In view of the importance of amide derivatives of pyrazoles, this paper reports the crystal structure of the title compound (I), C_19_H_19_N_3_O_2_.

The title compound, (I), crystallizes with two independent molecules in the asymmetric unit (A and B) (Fig. 1). In molecule A, the pyrazole ring adopts a slightly disordered half-chair conformation while in B it is planar. The dihedral angle between the mean planes of the two phenyl rings is 56.2 (8)° (A) and 38.2 (3)° (B). The N-phenyl substituent on the pyrazole ring is twisted by 46.5 (2)° (A) and 58.6 (4)° (B) while the extended phenyl ring is twisted by 82.2 (8)° (A) and 87.5 (9)° (B). The mean plane of the amide group forms an angle of 74.8 (3)° (A)(C2A/C1A/O1A/N1A), 67.7 (1)° (B)(C2B/C1B/O1B/N1B) with respect to that of the phenyl rings. In addition, the amide group is rotated by 51.4 (1)° (A), 53.6 (2)° (B) from the the mean plane of the pyrazole rings. Bond lengths are in normal ranges (Allen *et al.*, 1987). N—H···O intermolecular hydrogen bonds supported by a weak C14A—H14A···O1A intermolecular interaction are observed which link the molecules into dimers forming R_2_^2^(10) graph set motifs (Fig. 2). Also, additional weak C—H···O intermolecular interactions are also observed which interlink the dimers and influence the crystal packing.

### 2. Experimental

Phenylacetic acid (0.136 g, 1 mmol) and 4-aminoantipyrine (0.203 g, 1 mmol), 1-ethyl-3-(3-dimethylaminopropyl)-carbodiimide hydrochloride (1.0 g, 0.01 mol) and were dissolved in dichloromethane (20 mL). The mixture was stirred in presence of triethylamine at 273 K for about 3 h. The contents were poured into 100 ml of ice-cold aqueous hydrochloric acid with stirring, which was extracted thrice with dichloromethane. Organic layer was washed with saturated NaHCO_3_ solution and brine solution, dried and concentrated under reduced pressure to give the title compound (I). Single crystals were grown from methanol and acetone mixture (1:1) and further recrystallised from ethanol by by the slow evaporation method which were used as such for X-ray studies (M.P.: 445-447 K).

### 3. Refinement

All of the H atoms were placed in their calculated positions and then refined using the riding model with Atom—H lengths of 0.93Å (CH); 0.97Å (CH_2_); 0.96Å (CH_3_) or 0.86Å (NH). Isotropic displacement parameters for these atoms were set to 1.2 (CH, CH_2_, NH)and 1.5 (CH_3_) times *U*_eq_ of the parent atom. Idealised Me refined as rotating group.

### Crystal data

**Table:**

C_19_H_19_N_3_O_2_	*Z* = 4
*M**_r_* = 321.37	*F*(000) = 680
Triclinic, *P*1	*D*_x_ = 1.298 Mg m^−^^3^
*a* = 10.1258 (7) Å	Cu *K*α radiation, λ = 1.54184 Å
*b* = 10.4671 (8) Å	Cell parameters from 4663 reflections
*c* = 17.8888 (12) Å	θ = 4.9–72.3°
α = 100.833 (6)°	µ = 0.69 mm^−^^1^
β = 92.527 (5)°	*T* = 173 K
γ = 116.812 (7)°	Irregular, colourless
*V* = 1643.9 (2) Å^3^	0.48 × 0.32 × 0.26 mm

### Data collection

**Table:**

Agilent Xcalibur (Eos, Gemini) diffractometer	6333 independent reflections
Radiation source: Enhance (Cu) X-ray Source	5485 reflections with *I* > 2σ(*I*)
Detector resolution: 16.0416 pixels mm^-1^	*R*_int_ = 0.037
ω scans	θ_max_ = 72.4°, θ_min_ = 4.9°
Absorption correction: multi-scan (*CrysAlis PRO* and *CrysAlis RED*; Agilent, 2012)	*h* = −12→10
*T*_min_ = 0.876, *T*_max_ = 1.000	*k* = −12→12
10216 measured reflections	*l* = −17→21

### Refinement

**Table:**

Refinement on *F*^2^	Hydrogen site location: inferred from neighbouring sites
Least-squares matrix: full	H-atom parameters constrained
*R*[*F*^2^ > 2σ(*F*^2^)] = 0.046	*w* = 1/[σ^2^(*F*_o_^2^) + (0.0717*P*)^2^ + 0.280*P*] where *P* = (*F*_o_^2^ + 2*F*_c_^2^)/3
*wR*(*F*^2^) = 0.130	(Δ/σ)_max_ < 0.001
*S* = 1.04	Δρ_max_ = 0.30 e Å^−^^3^
6333 reflections	Δρ_min_ = −0.23 e Å^−^^3^
438 parameters	Extinction correction: *SHELXL*, Fc^*^=kFc[1+0.001xFc^2^λ^3^/sin(2θ)]^-1/4^
0 restraints	Extinction coefficient: 0.0080 (5)
Primary atom site location: structure-invariant direct methods	

### Special details

**Table:**

Geometry. All esds (except the esd in the dihedral angle between two l.s. planes) are estimated using the full covariance matrix. The cell esds are taken into account individually in the estimation of esds in distances, angles and torsion angles; correlations between esds in cell parameters are only used when they are defined by crystal symmetry. An approximate (isotropic) treatment of cell esds is used for estimating esds involving l.s. planes.

### Fractional atomic coordinates and isotropic or equivalent isotropic displacement parameters (Å^2^)

**Table:**

	*x*	*y*	*z*	*U*_iso_*/*U*_eq_	
O1A	0.53484 (12)	0.15316 (13)	0.11113 (6)	0.0325 (3)	
O2A	0.18670 (13)	0.29954 (12)	0.18901 (6)	0.0295 (3)	
N1A	0.30791 (13)	0.08641 (13)	0.15051 (7)	0.0224 (3)	
H1A	0.2487	0.0394	0.1800	0.027*	
N2A	0.16758 (14)	0.28576 (14)	0.05759 (7)	0.0241 (3)	
N3A	0.21484 (14)	0.22128 (14)	−0.00455 (7)	0.0249 (3)	
C1A	0.44894 (16)	0.10244 (16)	0.15614 (8)	0.0241 (3)	
C2A	0.49459 (18)	0.05345 (18)	0.22348 (10)	0.0309 (3)	
H2AA	0.4141	0.0214	0.2542	0.037*	
H2AB	0.5138	−0.0287	0.2043	0.037*	
C3A	0.63393 (18)	0.18034 (18)	0.27245 (9)	0.0291 (3)	
C4A	0.62384 (19)	0.2730 (2)	0.33564 (10)	0.0346 (4)	
H4A	0.5308	0.2514	0.3507	0.042*	
C5A	0.7501 (2)	0.3977 (2)	0.37709 (10)	0.0399 (4)	
H5A	0.7412	0.4589	0.4193	0.048*	
C6A	0.8889 (2)	0.4302 (2)	0.35513 (10)	0.0403 (4)	
H6A	0.9736	0.5140	0.3822	0.048*	
C7A	0.90161 (19)	0.3379 (2)	0.29293 (11)	0.0398 (4)	
H7A	0.9951	0.3591	0.2785	0.048*	
C8A	0.77540 (19)	0.2139 (2)	0.25202 (10)	0.0348 (4)	
H8A	0.7850	0.1522	0.2104	0.042*	
C9A	0.25487 (15)	0.14376 (15)	0.09827 (8)	0.0210 (3)	
C10A	0.25652 (16)	0.12695 (16)	0.02099 (8)	0.0236 (3)	
C11A	0.20064 (15)	0.24720 (15)	0.12389 (8)	0.0210 (3)	
C12A	0.17598 (16)	0.42661 (16)	0.05939 (8)	0.0241 (3)	
C13A	0.27423 (17)	0.52009 (18)	0.01869 (9)	0.0295 (3)	
H13A	0.3338	0.4912	−0.0107	0.035*	
C14A	0.2824 (2)	0.65726 (19)	0.02239 (10)	0.0374 (4)	
H14A	0.3457	0.7197	−0.0057	0.045*	
C15A	0.1962 (2)	0.70113 (19)	0.06787 (11)	0.0408 (4)	
H15A	0.2031	0.7937	0.0710	0.049*	
C16A	0.0999 (2)	0.6075 (2)	0.10855 (10)	0.0381 (4)	
H16A	0.0429	0.6378	0.1394	0.046*	
C17A	0.08765 (18)	0.46855 (18)	0.10377 (9)	0.0305 (3)	
H17A	0.0209	0.4046	0.1301	0.037*	
C18A	0.12208 (19)	0.17807 (18)	−0.07928 (8)	0.0312 (3)	
H18A	0.0243	0.1003	−0.0784	0.047*	
H18B	0.1675	0.1444	−0.1190	0.047*	
H18C	0.1137	0.2612	−0.0894	0.047*	
C19A	0.2914 (2)	0.02504 (19)	−0.03425 (9)	0.0345 (4)	
H19A	0.2002	−0.0542	−0.0638	0.052*	
H19B	0.3421	−0.0140	−0.0064	0.052*	
H19C	0.3545	0.0776	−0.0682	0.052*	
O1B	0.31592 (13)	0.34788 (13)	0.46337 (6)	0.0366 (3)	
O2B	0.09934 (12)	−0.05612 (12)	0.24555 (6)	0.0285 (3)	
N1B	0.21077 (13)	0.25496 (13)	0.33811 (7)	0.0233 (3)	
H1B	0.2207	0.2679	0.2922	0.028*	
N2B	−0.08803 (13)	−0.10989 (13)	0.32306 (7)	0.0240 (3)	
N3B	−0.13148 (14)	−0.02662 (14)	0.37736 (7)	0.0257 (3)	
C1B	0.31460 (16)	0.35901 (16)	0.39659 (8)	0.0243 (3)	
C2B	0.42779 (16)	0.49491 (16)	0.37249 (9)	0.0268 (3)	
H2BA	0.5238	0.5348	0.4044	0.032*	
H2BB	0.4406	0.4673	0.3195	0.032*	
C3B	0.37614 (16)	0.61150 (16)	0.38034 (9)	0.0247 (3)	
C4B	0.40330 (17)	0.70709 (18)	0.45149 (9)	0.0302 (3)	
H4B	0.4505	0.6971	0.4942	0.036*	
C5B	0.3608 (2)	0.8167 (2)	0.45926 (11)	0.0384 (4)	
H5B	0.3791	0.8796	0.5071	0.046*	
C6B	0.2910 (2)	0.8332 (2)	0.39606 (11)	0.0398 (4)	
H6B	0.2640	0.9081	0.4013	0.048*	
C7B	0.26169 (19)	0.7381 (2)	0.32530 (11)	0.0366 (4)	
H7B	0.2141	0.7482	0.2828	0.044*	
C8B	0.30353 (17)	0.62717 (17)	0.31766 (9)	0.0293 (3)	
H8B	0.2827	0.5627	0.2700	0.035*	
C9B	0.08712 (15)	0.12641 (16)	0.34769 (8)	0.0219 (3)	
C10B	−0.01869 (16)	0.11522 (16)	0.39461 (8)	0.0239 (3)	
C11B	0.04395 (15)	−0.01523 (16)	0.29956 (8)	0.0216 (3)	
C12B	−0.20372 (16)	−0.23775 (16)	0.27073 (8)	0.0247 (3)	
C13B	−0.17998 (19)	−0.35804 (17)	0.24651 (9)	0.0313 (3)	
H13B	−0.0928	−0.3574	0.2658	0.038*	
C14B	−0.2880 (2)	−0.48003 (18)	0.19304 (10)	0.0404 (4)	
H14B	−0.2729	−0.5614	0.1762	0.049*	
C15B	−0.4170 (2)	−0.4809 (2)	0.16501 (10)	0.0443 (5)	
H15B	−0.4885	−0.5625	0.1289	0.053*	
C16B	−0.44110 (19)	−0.3609 (2)	0.19021 (10)	0.0426 (5)	
H16B	−0.5291	−0.3627	0.1714	0.051*	
C17B	−0.33419 (18)	−0.23789 (19)	0.24349 (10)	0.0332 (4)	
H17B	−0.3498	−0.1569	0.2606	0.040*	
C18B	−0.2191 (2)	−0.09991 (19)	0.43300 (10)	0.0359 (4)	
H18D	−0.2967	−0.1957	0.4069	0.054*	
H18E	−0.2632	−0.0427	0.4584	0.054*	
H18F	−0.1553	−0.1097	0.4703	0.054*	
C19B	−0.02449 (19)	0.22860 (18)	0.45642 (9)	0.0327 (4)	
H19D	−0.1216	0.2233	0.4501	0.049*	
H19E	0.0502	0.3245	0.4534	0.049*	
H19F	−0.0059	0.2112	0.5057	0.049*	

### Atomic displacement parameters (Å^2^)

**Table:**

	*U*^11^	*U*^22^	*U*^33^	*U*^12^	*U*^13^	*U*^23^
O1A	0.0297 (6)	0.0411 (7)	0.0298 (6)	0.0174 (5)	0.0106 (5)	0.0121 (5)
O2A	0.0455 (6)	0.0349 (6)	0.0183 (5)	0.0265 (5)	0.0104 (4)	0.0078 (4)
N1A	0.0257 (6)	0.0216 (6)	0.0227 (6)	0.0116 (5)	0.0069 (5)	0.0091 (5)
N2A	0.0318 (6)	0.0277 (6)	0.0178 (6)	0.0176 (5)	0.0064 (5)	0.0060 (5)
N3A	0.0320 (6)	0.0281 (6)	0.0161 (6)	0.0161 (5)	0.0050 (5)	0.0032 (5)
C1A	0.0270 (7)	0.0215 (7)	0.0251 (7)	0.0124 (6)	0.0051 (6)	0.0050 (5)
C2A	0.0333 (8)	0.0311 (8)	0.0355 (9)	0.0186 (7)	0.0066 (7)	0.0138 (7)
C3A	0.0333 (8)	0.0364 (8)	0.0272 (8)	0.0216 (7)	0.0060 (6)	0.0146 (6)
C4A	0.0362 (9)	0.0449 (10)	0.0313 (8)	0.0242 (8)	0.0098 (7)	0.0132 (7)
C5A	0.0509 (10)	0.0450 (10)	0.0282 (8)	0.0268 (9)	0.0049 (7)	0.0070 (7)
C6A	0.0390 (9)	0.0442 (10)	0.0334 (9)	0.0156 (8)	−0.0042 (7)	0.0118 (8)
C7A	0.0299 (8)	0.0564 (11)	0.0387 (9)	0.0223 (8)	0.0058 (7)	0.0182 (8)
C8A	0.0365 (9)	0.0486 (10)	0.0293 (8)	0.0272 (8)	0.0086 (7)	0.0116 (7)
C9A	0.0211 (6)	0.0201 (7)	0.0198 (7)	0.0082 (5)	0.0035 (5)	0.0038 (5)
C10A	0.0260 (7)	0.0208 (7)	0.0220 (7)	0.0099 (6)	0.0047 (5)	0.0032 (5)
C11A	0.0218 (6)	0.0220 (7)	0.0193 (7)	0.0094 (6)	0.0039 (5)	0.0066 (5)
C12A	0.0287 (7)	0.0255 (7)	0.0192 (7)	0.0140 (6)	−0.0006 (6)	0.0052 (5)
C13A	0.0301 (8)	0.0313 (8)	0.0253 (7)	0.0126 (7)	0.0042 (6)	0.0075 (6)
C14A	0.0424 (9)	0.0276 (8)	0.0354 (9)	0.0092 (7)	0.0026 (7)	0.0117 (7)
C15A	0.0582 (11)	0.0285 (8)	0.0371 (9)	0.0233 (8)	−0.0028 (8)	0.0047 (7)
C16A	0.0542 (11)	0.0437 (10)	0.0297 (8)	0.0353 (9)	0.0056 (8)	0.0056 (7)
C17A	0.0372 (8)	0.0364 (9)	0.0249 (7)	0.0216 (7)	0.0067 (6)	0.0108 (6)
C18A	0.0365 (8)	0.0350 (8)	0.0190 (7)	0.0146 (7)	0.0013 (6)	0.0055 (6)
C19A	0.0485 (10)	0.0343 (9)	0.0239 (8)	0.0237 (8)	0.0086 (7)	0.0018 (6)
O1B	0.0345 (6)	0.0386 (6)	0.0229 (6)	0.0053 (5)	−0.0029 (5)	0.0095 (5)
O2B	0.0286 (5)	0.0264 (5)	0.0278 (6)	0.0105 (4)	0.0117 (4)	0.0049 (4)
N1B	0.0244 (6)	0.0222 (6)	0.0190 (6)	0.0064 (5)	0.0040 (5)	0.0072 (5)
N2B	0.0220 (6)	0.0233 (6)	0.0228 (6)	0.0076 (5)	0.0066 (5)	0.0036 (5)
N3B	0.0255 (6)	0.0255 (6)	0.0236 (6)	0.0096 (5)	0.0096 (5)	0.0052 (5)
C1B	0.0239 (7)	0.0240 (7)	0.0242 (7)	0.0103 (6)	0.0039 (5)	0.0064 (6)
C2B	0.0229 (7)	0.0243 (7)	0.0291 (8)	0.0075 (6)	0.0059 (6)	0.0056 (6)
C3B	0.0194 (6)	0.0214 (7)	0.0282 (7)	0.0044 (5)	0.0088 (6)	0.0067 (6)
C4B	0.0259 (7)	0.0326 (8)	0.0269 (8)	0.0099 (6)	0.0064 (6)	0.0049 (6)
C5B	0.0379 (9)	0.0342 (9)	0.0370 (9)	0.0146 (7)	0.0117 (7)	−0.0011 (7)
C6B	0.0420 (9)	0.0337 (9)	0.0510 (11)	0.0224 (8)	0.0169 (8)	0.0113 (8)
C7B	0.0358 (9)	0.0403 (9)	0.0389 (9)	0.0193 (8)	0.0094 (7)	0.0162 (7)
C8B	0.0293 (8)	0.0281 (8)	0.0263 (8)	0.0102 (6)	0.0059 (6)	0.0052 (6)
C9B	0.0220 (7)	0.0233 (7)	0.0192 (6)	0.0093 (6)	0.0031 (5)	0.0060 (5)
C10B	0.0245 (7)	0.0240 (7)	0.0219 (7)	0.0101 (6)	0.0032 (5)	0.0061 (6)
C11B	0.0205 (6)	0.0243 (7)	0.0202 (7)	0.0097 (6)	0.0029 (5)	0.0080 (5)
C12B	0.0237 (7)	0.0229 (7)	0.0218 (7)	0.0051 (6)	0.0060 (6)	0.0071 (6)
C13B	0.0335 (8)	0.0274 (8)	0.0305 (8)	0.0109 (7)	0.0073 (6)	0.0092 (6)
C14B	0.0487 (10)	0.0235 (8)	0.0364 (9)	0.0068 (7)	0.0137 (8)	0.0032 (7)
C15B	0.0340 (9)	0.0394 (10)	0.0287 (9)	−0.0058 (8)	0.0070 (7)	−0.0010 (7)
C16B	0.0242 (8)	0.0578 (12)	0.0300 (9)	0.0078 (8)	0.0023 (7)	0.0053 (8)
C17B	0.0289 (8)	0.0372 (9)	0.0298 (8)	0.0127 (7)	0.0062 (6)	0.0065 (7)
C18B	0.0373 (9)	0.0345 (9)	0.0339 (9)	0.0125 (7)	0.0183 (7)	0.0119 (7)
C19B	0.0365 (8)	0.0315 (8)	0.0298 (8)	0.0168 (7)	0.0095 (7)	0.0031 (6)

### Geometric parameters (Å, º)

**Table:**

O1A—C1A	1.2217 (18)	O1B—C1B	1.2219 (19)
O2A—C11A	1.2334 (17)	O2B—C11B	1.2412 (18)
N1A—H1A	0.8600	N1B—H1B	0.8600
N1A—C1A	1.3574 (19)	N1B—C1B	1.3519 (19)
N1A—C9A	1.4069 (18)	N1B—C9B	1.4118 (18)
N2A—N3A	1.4057 (16)	N2B—N3B	1.4008 (17)
N2A—C11A	1.3958 (18)	N2B—C11B	1.3985 (18)
N2A—C12A	1.4320 (19)	N2B—C12B	1.4345 (18)
N3A—C10A	1.375 (2)	N3B—C10B	1.3678 (19)
N3A—C18A	1.4673 (19)	N3B—C18B	1.4553 (19)
C1A—C2A	1.525 (2)	C1B—C2B	1.525 (2)
C2A—H2AA	0.9700	C2B—H2BA	0.9700
C2A—H2AB	0.9700	C2B—H2BB	0.9700
C2A—C3A	1.512 (2)	C2B—C3B	1.517 (2)
C3A—C4A	1.384 (2)	C3B—C4B	1.392 (2)
C3A—C8A	1.396 (2)	C3B—C8B	1.389 (2)
C4A—H4A	0.9300	C4B—H4B	0.9300
C4A—C5A	1.391 (3)	C4B—C5B	1.383 (2)
C5A—H5A	0.9300	C5B—H5B	0.9300
C5A—C6A	1.383 (3)	C5B—C6B	1.386 (3)
C6A—H6A	0.9300	C6B—H6B	0.9300
C6A—C7A	1.382 (3)	C6B—C7B	1.381 (3)
C7A—H7A	0.9300	C7B—H7B	0.9300
C7A—C8A	1.384 (3)	C7B—C8B	1.391 (2)
C8A—H8A	0.9300	C8B—H8B	0.9300
C9A—C10A	1.362 (2)	C9B—C10B	1.367 (2)
C9A—C11A	1.4339 (19)	C9B—C11B	1.426 (2)
C10A—C19A	1.488 (2)	C10B—C19B	1.486 (2)
C12A—C13A	1.389 (2)	C12B—C13B	1.381 (2)
C12A—C17A	1.382 (2)	C12B—C17B	1.387 (2)
C13A—H13A	0.9300	C13B—H13B	0.9300
C13A—C14A	1.389 (2)	C13B—C14B	1.389 (2)
C14A—H14A	0.9300	C14B—H14B	0.9300
C14A—C15A	1.385 (3)	C14B—C15B	1.374 (3)
C15A—H15A	0.9300	C15B—H15B	0.9300
C15A—C16A	1.381 (3)	C15B—C16B	1.383 (3)
C16A—H16A	0.9300	C16B—H16B	0.9300
C16A—C17A	1.389 (2)	C16B—C17B	1.389 (2)
C17A—H17A	0.9300	C17B—H17B	0.9300
C18A—H18A	0.9600	C18B—H18D	0.9600
C18A—H18B	0.9600	C18B—H18E	0.9600
C18A—H18C	0.9600	C18B—H18F	0.9600
C19A—H19A	0.9600	C19B—H19D	0.9600
C19A—H19B	0.9600	C19B—H19E	0.9600
C19A—H19C	0.9600	C19B—H19F	0.9600
			
C1A—N1A—H1A	118.7	C1B—N1B—H1B	117.9
C1A—N1A—C9A	122.53 (12)	C1B—N1B—C9B	124.10 (12)
C9A—N1A—H1A	118.7	C9B—N1B—H1B	117.9
N3A—N2A—C12A	118.71 (12)	N3B—N2B—C12B	117.75 (11)
C11A—N2A—N3A	109.01 (11)	C11B—N2B—N3B	109.03 (11)
C11A—N2A—C12A	122.24 (12)	C11B—N2B—C12B	122.09 (12)
N2A—N3A—C18A	114.99 (12)	N2B—N3B—C18B	116.82 (12)
C10A—N3A—N2A	107.00 (11)	C10B—N3B—N2B	107.38 (11)
C10A—N3A—C18A	121.64 (12)	C10B—N3B—C18B	124.49 (13)
O1A—C1A—N1A	123.06 (14)	O1B—C1B—N1B	123.34 (14)
O1A—C1A—C2A	121.64 (14)	O1B—C1B—C2B	122.55 (14)
N1A—C1A—C2A	115.30 (13)	N1B—C1B—C2B	114.08 (13)
C1A—C2A—H2AA	109.8	C1B—C2B—H2BA	109.5
C1A—C2A—H2AB	109.8	C1B—C2B—H2BB	109.5
H2AA—C2A—H2AB	108.2	H2BA—C2B—H2BB	108.0
C3A—C2A—C1A	109.43 (13)	C3B—C2B—C1B	110.89 (12)
C3A—C2A—H2AA	109.8	C3B—C2B—H2BA	109.5
C3A—C2A—H2AB	109.8	C3B—C2B—H2BB	109.5
C4A—C3A—C2A	120.74 (14)	C4B—C3B—C2B	119.81 (14)
C4A—C3A—C8A	118.09 (16)	C8B—C3B—C2B	121.54 (14)
C8A—C3A—C2A	121.00 (15)	C8B—C3B—C4B	118.65 (15)
C3A—C4A—H4A	119.3	C3B—C4B—H4B	119.7
C3A—C4A—C5A	121.35 (16)	C5B—C4B—C3B	120.61 (16)
C5A—C4A—H4A	119.3	C5B—C4B—H4B	119.7
C4A—C5A—H5A	120.2	C4B—C5B—H5B	119.8
C6A—C5A—C4A	119.60 (17)	C4B—C5B—C6B	120.31 (16)
C6A—C5A—H5A	120.2	C6B—C5B—H5B	119.8
C5A—C6A—H6A	120.0	C5B—C6B—H6B	120.1
C7A—C6A—C5A	119.93 (17)	C7B—C6B—C5B	119.74 (16)
C7A—C6A—H6A	120.0	C7B—C6B—H6B	120.1
C6A—C7A—H7A	120.0	C6B—C7B—H7B	120.0
C6A—C7A—C8A	120.08 (16)	C6B—C7B—C8B	119.90 (17)
C8A—C7A—H7A	120.0	C8B—C7B—H7B	120.0
C3A—C8A—H8A	119.5	C3B—C8B—C7B	120.78 (15)
C7A—C8A—C3A	120.94 (16)	C3B—C8B—H8B	119.6
C7A—C8A—H8A	119.5	C7B—C8B—H8B	119.6
N1A—C9A—C11A	121.51 (12)	N1B—C9B—C11B	122.38 (12)
C10A—C9A—N1A	129.66 (13)	C10B—C9B—N1B	128.32 (13)
C10A—C9A—C11A	108.58 (13)	C10B—C9B—C11B	108.85 (13)
N3A—C10A—C19A	120.26 (13)	N3B—C10B—C19B	120.50 (13)
C9A—C10A—N3A	109.52 (13)	C9B—C10B—N3B	109.22 (13)
C9A—C10A—C19A	130.20 (14)	C9B—C10B—C19B	130.28 (14)
O2A—C11A—N2A	123.95 (13)	O2B—C11B—N2B	123.28 (13)
O2A—C11A—C9A	130.81 (13)	O2B—C11B—C9B	131.65 (13)
N2A—C11A—C9A	105.21 (12)	N2B—C11B—C9B	105.02 (12)
C13A—C12A—N2A	120.23 (13)	C13B—C12B—N2B	118.52 (14)
C17A—C12A—N2A	118.71 (13)	C13B—C12B—C17B	121.12 (14)
C17A—C12A—C13A	121.04 (14)	C17B—C12B—N2B	120.34 (14)
C12A—C13A—H13A	120.4	C12B—C13B—H13B	120.4
C14A—C13A—C12A	119.21 (15)	C12B—C13B—C14B	119.18 (16)
C14A—C13A—H13A	120.4	C14B—C13B—H13B	120.4
C13A—C14A—H14A	120.0	C13B—C14B—H14B	119.9
C15A—C14A—C13A	120.06 (16)	C15B—C14B—C13B	120.24 (17)
C15A—C14A—H14A	120.0	C15B—C14B—H14B	119.9
C14A—C15A—H15A	120.0	C14B—C15B—H15B	119.8
C16A—C15A—C14A	120.08 (16)	C14B—C15B—C16B	120.34 (16)
C16A—C15A—H15A	120.0	C16B—C15B—H15B	119.8
C15A—C16A—H16A	119.8	C15B—C16B—H16B	119.9
C15A—C16A—C17A	120.48 (16)	C15B—C16B—C17B	120.20 (17)
C17A—C16A—H16A	119.8	C17B—C16B—H16B	119.9
C12A—C17A—C16A	119.08 (15)	C12B—C17B—C16B	118.91 (17)
C12A—C17A—H17A	120.5	C12B—C17B—H17B	120.5
C16A—C17A—H17A	120.5	C16B—C17B—H17B	120.5
N3A—C18A—H18A	109.5	N3B—C18B—H18D	109.5
N3A—C18A—H18B	109.5	N3B—C18B—H18E	109.5
N3A—C18A—H18C	109.5	N3B—C18B—H18F	109.5
H18A—C18A—H18B	109.5	H18D—C18B—H18E	109.5
H18A—C18A—H18C	109.5	H18D—C18B—H18F	109.5
H18B—C18A—H18C	109.5	H18E—C18B—H18F	109.5
C10A—C19A—H19A	109.5	C10B—C19B—H19D	109.5
C10A—C19A—H19B	109.5	C10B—C19B—H19E	109.5
C10A—C19A—H19C	109.5	C10B—C19B—H19F	109.5
H19A—C19A—H19B	109.5	H19D—C19B—H19E	109.5
H19A—C19A—H19C	109.5	H19D—C19B—H19F	109.5
H19B—C19A—H19C	109.5	H19E—C19B—H19F	109.5
			
O1A—C1A—C2A—C3A	56.40 (19)	O1B—C1B—C2B—C3B	−86.23 (18)
N1A—C1A—C2A—C3A	−123.19 (14)	N1B—C1B—C2B—C3B	91.80 (15)
N1A—C9A—C10A—N3A	−170.40 (14)	N1B—C9B—C10B—N3B	170.33 (14)
N1A—C9A—C10A—C19A	11.1 (3)	N1B—C9B—C10B—C19B	−9.8 (3)
N1A—C9A—C11A—O2A	−1.8 (2)	N1B—C9B—C11B—O2B	1.9 (2)
N1A—C9A—C11A—N2A	176.26 (12)	N1B—C9B—C11B—N2B	−175.40 (12)
N2A—N3A—C10A—C9A	−7.50 (16)	N2B—N3B—C10B—C9B	5.75 (16)
N2A—N3A—C10A—C19A	171.13 (13)	N2B—N3B—C10B—C19B	−174.13 (13)
N2A—C12A—C13A—C14A	−178.87 (14)	N2B—C12B—C13B—C14B	−176.98 (14)
N2A—C12A—C17A—C16A	177.18 (14)	N2B—C12B—C17B—C16B	177.11 (14)
N3A—N2A—C11A—O2A	172.11 (13)	N3B—N2B—C11B—O2B	−171.57 (13)
N3A—N2A—C11A—C9A	−6.14 (15)	N3B—N2B—C11B—C9B	6.02 (15)
N3A—N2A—C12A—C13A	−22.75 (19)	N3B—N2B—C12B—C13B	−146.60 (14)
N3A—N2A—C12A—C17A	158.89 (13)	N3B—N2B—C12B—C17B	35.38 (19)
C1A—N1A—C9A—C10A	52.2 (2)	C1B—N1B—C9B—C10B	53.7 (2)
C1A—N1A—C9A—C11A	−121.29 (15)	C1B—N1B—C9B—C11B	−134.85 (15)
C1A—C2A—C3A—C4A	93.50 (17)	C1B—C2B—C3B—C4B	83.37 (16)
C1A—C2A—C3A—C8A	−81.70 (18)	C1B—C2B—C3B—C8B	−97.55 (16)
C2A—C3A—C4A—C5A	−174.17 (15)	C2B—C3B—C4B—C5B	178.16 (14)
C2A—C3A—C8A—C7A	174.12 (16)	C2B—C3B—C8B—C7B	−177.67 (14)
C3A—C4A—C5A—C6A	−0.2 (3)	C3B—C4B—C5B—C6B	−0.3 (3)
C4A—C3A—C8A—C7A	−1.2 (2)	C4B—C3B—C8B—C7B	1.4 (2)
C4A—C5A—C6A—C7A	−0.8 (3)	C4B—C5B—C6B—C7B	1.1 (3)
C5A—C6A—C7A—C8A	0.8 (3)	C5B—C6B—C7B—C8B	−0.6 (3)
C6A—C7A—C8A—C3A	0.3 (3)	C6B—C7B—C8B—C3B	−0.7 (2)
C8A—C3A—C4A—C5A	1.2 (2)	C8B—C3B—C4B—C5B	−1.0 (2)
C9A—N1A—C1A—O1A	−8.2 (2)	C9B—N1B—C1B—O1B	4.1 (2)
C9A—N1A—C1A—C2A	171.37 (13)	C9B—N1B—C1B—C2B	−173.89 (13)
C10A—C9A—C11A—O2A	−176.54 (15)	C10B—C9B—C11B—O2B	174.80 (15)
C10A—C9A—C11A—N2A	1.55 (15)	C10B—C9B—C11B—N2B	−2.50 (16)
C11A—N2A—N3A—C10A	8.50 (15)	C11B—N2B—N3B—C10B	−7.40 (16)
C11A—N2A—N3A—C18A	146.89 (13)	C11B—N2B—N3B—C18B	−152.81 (13)
C11A—N2A—C12A—C13A	118.93 (16)	C11B—N2B—C12B—C13B	73.50 (18)
C11A—N2A—C12A—C17A	−59.42 (19)	C11B—N2B—C12B—C17B	−104.52 (17)
C11A—C9A—C10A—N3A	3.74 (16)	C11B—C9B—C10B—N3B	−2.02 (17)
C11A—C9A—C10A—C19A	−174.72 (15)	C11B—C9B—C10B—C19B	177.85 (15)
C12A—N2A—N3A—C10A	154.81 (12)	C12B—N2B—N3B—C10B	−152.14 (13)
C12A—N2A—N3A—C18A	−66.80 (17)	C12B—N2B—N3B—C18B	62.45 (18)
C12A—N2A—C11A—O2A	27.2 (2)	C12B—N2B—C11B—O2B	−28.7 (2)
C12A—N2A—C11A—C9A	−151.03 (13)	C12B—N2B—C11B—C9B	148.94 (13)
C12A—C13A—C14A—C15A	1.7 (2)	C12B—C13B—C14B—C15B	−0.3 (2)
C13A—C12A—C17A—C16A	−1.2 (2)	C13B—C12B—C17B—C16B	−0.9 (2)
C13A—C14A—C15A—C16A	−1.2 (3)	C13B—C14B—C15B—C16B	−0.5 (3)
C14A—C15A—C16A—C17A	−0.6 (3)	C14B—C15B—C16B—C17B	0.7 (3)
C15A—C16A—C17A—C12A	1.7 (3)	C15B—C16B—C17B—C12B	0.0 (3)
C17A—C12A—C13A—C14A	−0.6 (2)	C17B—C12B—C13B—C14B	1.0 (2)
C18A—N3A—C10A—C9A	−142.51 (14)	C18B—N3B—C10B—C9B	147.82 (15)
C18A—N3A—C10A—C19A	36.1 (2)	C18B—N3B—C10B—C19B	−32.1 (2)

### Hydrogen-bond geometry (Å, º)

**Table:**

*D*—H···*A*	*D*—H	H···*A*	*D*···*A*	*D*—H···*A*
N1*A*—H1*A*···O2*B*	0.86	1.97	2.8292 (16)	173
C14*A*—H14*A*···O1*A*^i^	0.93	2.55	3.454 (2)	165
N1*B*—H1*B*···O2*A*	0.86	1.98	2.8115 (16)	163
C2*B*—H2*BA*···O1*B*^ii^	0.97	2.55	3.4239 (19)	150
C4*B*—H4*B*···O1*B*^ii^	0.93	2.72	3.487 (2)	141
C8*B*—H8*B*···O2*A*	0.93	2.57	3.404 (2)	150
C14*B*—H14*B*···O1*A*^iii^	0.93	2.70	3.398 (2)	132

Symmetry codes: (i) −*x*+1, −*y*+1, −*z*; (ii) −*x*+1, −*y*+1, −*z*+1; (iii) *x*−1, *y*−1, *z*.
